# Exploring the comorbidity of type 2 diabetes mellitus and polycystic ovary syndrome: treatment with traditional Chinese medicine

**DOI:** 10.3389/fmed.2026.1764278

**Published:** 2026-05-25

**Authors:** Tianjiao Li, Shuyi Zhao, Xue Zhou, Mingyue Kong, Ziqi Dai, Xin Chen

**Affiliations:** 1 Department of Gynecology, Changchun University of Chinese Medicine, Changchun, Jilin, China; 2 Department of Obstetrics, Affiliated Hospital of Changchun University of Chinese Medicine, Changchun, Jilin, China; 3 Department of Gynecology, The Affiliated Hospital to Changchun University of Chinese Medicine, Changchun, Jilin, China

**Keywords:** comorbidity, diagnosis, pathological mechanism, polycystic ovary syndrome, traditional Chinese medicine, treatment, type 2 diabetes mellitus

## Abstract

Type 2 diabetes mellitus (T2DM) and polycystic ovary syndrome (PCOS) are prevalent clinical conditions that frequently coexist and exhibit intricate pathophysiological interrelationships and shared genetic susceptibilities. T2DM is primarily characterized by insulin resistance and β-cell dysfunction, whereas PCOS, a common endocrine disorder among women of reproductive age, presents with multiple phenotypic variants, with the classical phenotype conferring the highest risk of T2DM development. Epidemiological studies indicate that women with PCOS have a significantly increased risk of developing T2DM, particularly in the presence of obesity, hyperandrogenism, and other metabolic risk factors. The coexistence of these two disorders reflects overlapping endocrine and metabolic abnormalities and is associated with increased long-term health burden. Diagnosis requires integrated assessment of glucose metabolism, reproductive features, and related clinical indicators. Current management includes lifestyle intervention, pharmacological treatment, and selected surgical approaches, while traditional Chinese medicine may provide complementary value in symptom regulation and metabolic improvement. However, further high-quality studies are needed to clarify its efficacy and mechanisms. Future research should focus on standardized diagnostic and therapeutic strategies and on strengthening evidence for integrated management.

## Introduction

Type 2 diabetes mellitus (T2DM) and polycystic ovary syndrome (PCOS) are two common endocrine-metabolic disorders that frequently coexist in clinical practice. Increasing evidence indicates that women with PCOS are at elevated risk of impaired glucose metabolism and subsequent development of T2DM (1–3). T2DM is primarily characterized by insulin resistance and progressive pancreatic β-cell dysfunction (1), whereas PCOS represents the most common endocrine disorder among women of reproductive age, with a heterogeneous clinical presentation involving reproductive, metabolic, and endocrine abnormalities (2, 3). PCOS is usually categorized into four phenotypes, and among these, the classical phenotype is characterized by both ovulatory dysfunction and hyperandrogenism and has been consistently associated with the highest risk of progression to T2DM (4, 5).

From a clinical perspective, the coexistence of T2DM and PCOS indicates a convergence of metabolic and reproductive dysfunction (5). Epidemiological studies reported that women with PCOS exhibit a significantly increased incidence of T2DM compared with the general population, with risk further increased by obesity, hyperandrogenism, genetic predisposition, and lifestyle factors (6). T2DM tends to develop at a younger age in patients with PCOS, often occurring 10–15 years earlier than in individuals without PCOS. Clinically, the diagnosis of PCOS in women with T2DM requires a comprehensive evaluation integrating metabolic and reproductive features (7), with particular caution required in adolescents, in whom both hyperandrogenism and ovulatory dysfunction are required, while ultrasound and AMH are not recommended for diagnosis. Furthermore, patients with concurrent T2DM and PCOS frequently present with additional comorbidities, including non-alcoholic fatty liver disease (NAFLD) and obstructive sleep apnea (OSA), which further complicate disease management (8, 9).

Given the increasing prevalence and clinical complexity of T2DM combined with PCOS, a comprehensive understanding of their shared and distinct characteristics is essential. Herein, this review aims to discuss the epidemiology, pathophysiological mechanisms, genetic basis, diagnostic strategies, and therapeutic approaches of this comorbidity, with particular emphasis on the integrative role of traditional Chinese medicine (TCM) with medical management. By merging current evidence, we aim to provide a referential guide for clinical decision-making and potential future research in this evolving field. The literature search for this review is provided in the Supplementary material.

## Epidemiology of PCOS in T2DM

The global prevalence of PCOS ranges from approximately 5%–18%, with a concomitant incidence of T2DM reported between 10% and 30%, markedly exceeding the 5%–10% observed in the general population (10). A meta-analysis encompassing 10,074 individuals with PCOS revealed a relative risk (RR) of 3.45 for developing T2DM, with obese patients exhibiting an elevated RR of 4.86, compared to 1.62 in non-obese counterparts (11). The peak incidence of T2DM among PCOS patients occurs between 35 and 45 years of age, which is 10–15 years earlier than that in the general population (12). Notably, the prevalence of T2DM in South Asian women with PCOS is 8.1%, surpassing that observed in white populations (13).

The prevalence of MS among individuals with both T2DM and PCOS is notably high, ranging from 60% to 80%, with hypertension, dyslipidemia, and central obesity affecting 40%–50%, 50%–60%, and 70%–80% of patients, respectively (14, 15). Furthermore, cardiovascular disease risk was significantly elevated in this cohort, and a prospective cohort study reported an incidence rate of coronary heart disease of 2.47 per 1,000 person-years in PCOS patients, representing a 2.3-fold increase relative to that in women without PCOS (16). The risk of cerebrovascular events is also elevated by 1.5–2 times and correlates positively with the severity of insulin resistance (17). Additionally, PCOS patients exhibit a higher prevalence of mental health disorders, with depression and anxiety affecting 30%–40% and 20%–30% of patients, respectively, which may further exacerbate metabolic dysfunction (17, 18).

Geographical disparities in the prevalence of T2DM among PCOS patients are evident globally, with the highest rates reported in the Middle East (25%–30%), followed by South Asia (20%–25%), Europe (15%–20%), and East Asia (10%–15%) (3, 6). Data from the Danish National Registry indicate a 7.5% prevalence of T2DM among 19,199 PCOS patients, which is 2.5 times greater than that in age-matched controls (17). Similarly, a multicenter study in China documented a T2DM prevalence of 10.8% in PCOS patients, including 18.2% among those classified as obese (19).

Age-stratified analyses demonstrated a low prevalence of T2DM in adolescent PCOS patients (approximately 2%–5%), with a marked increase observed with advancing age: 8%–12% in individuals aged 20–30 years, 15%–20% in those aged 30–40 years, and 25%–30% in patients aged >40 years (12, 19). Regarding familial risk, male relatives of PCOS patients also exhibit increased susceptibility; men with a sister diagnosed with PCOS have a 1.62-fold (95% CI: 1.34–1.96) increased risk of developing T2DM (20). Moreover, during the COVID-19 pandemic, the incidence of T2DM among PCOS patients has increased by 2.28 times, potentially attributable to sedentary behavior and elevated psychological stress (3).

Obesity is the most significant risk factor for T2DM in PCOS. Patients with a body mass index (BMI) ≥ 25 kg/m^2^ have a 3.85-fold increased risk compared with those with a BMI < 25 kg/m^2^ (11, 21). Central obesity exerts an even greater effect; PCOS patients with a waist circumference ≥88 cm demonstrate a 4.2-fold increased risk of T2DM, which correlates positively with insulin resistance (22, 23). The hyperandrogenic phenotype also represents a critical risk factor, with PCOS patients exhibiting free testosterone levels ≥4.5 pg/mL experiencing a 3.86-fold increased risk of T2DM compared to those without this phenotype (4).

**FIGURE 1:**
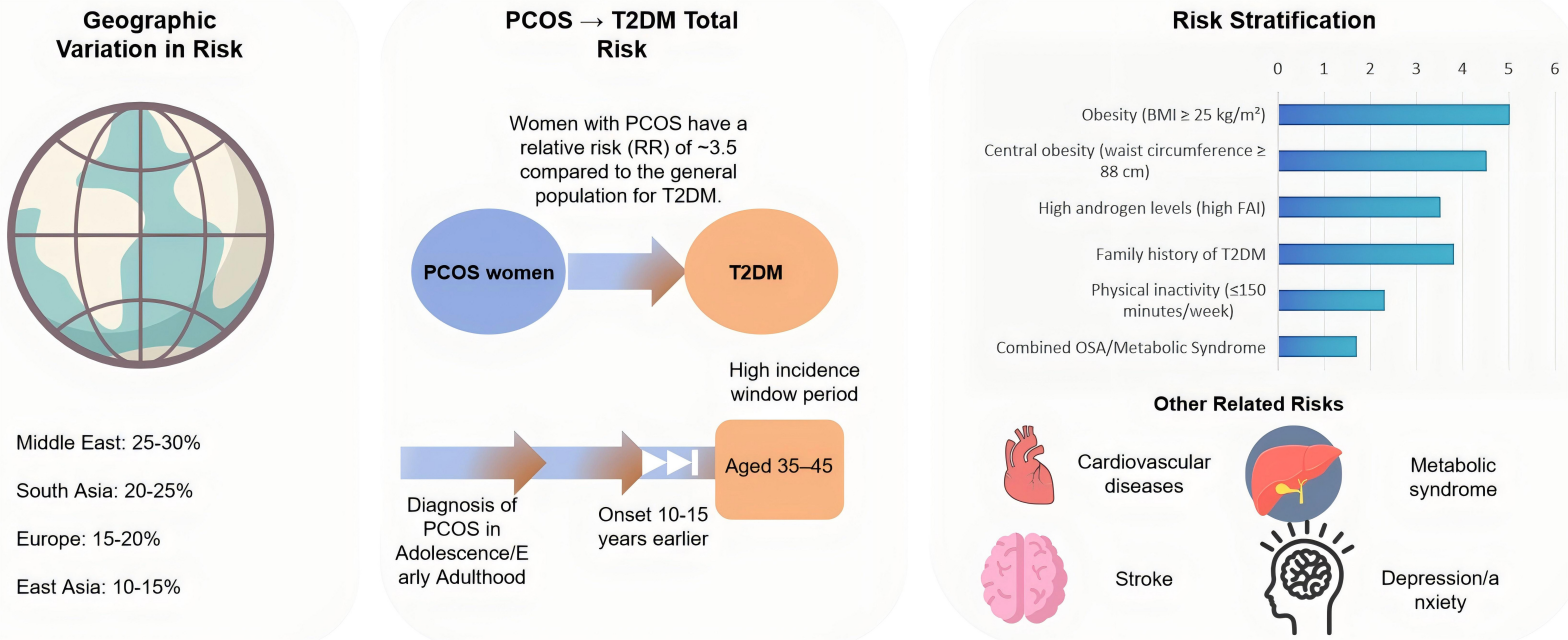
Geographic variations and stratified risks of total risk of type 2 diabetes mellitus (T2DM) in women with polycystic ovary syndrome (PCOS). This figure illustrates the global geographic variation in the risk of T2DM in women with PCOS and the stratified risks based on various factors. The first section highlights the geographic variation in T2DM risk, showing different prevalence rates across regions: Middle East (25%–30%), South Asia (20%–25%), Europe (15%–20%), and East Asia (10%-15%). The second part emphasizes the increased relative risk (RR ∼3.5) of T2DM in women with PCOS compared to the general population. It also depicts the early onset of T2DM in PCOS patients, typically 10–15 years earlier than the general population, especially in women aged 35–45. The last section illustrates risk stratification based on obesity, central obesity, high androgen levels, family history, physical inactivity, and metabolic syndrome, highlighting their contribution to T2DM risk in PCOS women.

Genetic predisposition further influences T2DM risk in patients with PCOS. Those with first-degree relatives affected by T2DM have a 2.3-fold increased risk (19), and carriers of the TCF7L2 rs7903146 T allele exhibit a 1.7-fold elevated risk (24). Lifestyle factors also contribute significantly; sedentary behavior (defined as less than 150 min of exercise per week) is associated with a 2.1-fold increased risk, while a high-carbohydrate diet (added sugar intake ≥ 25 g/day) corresponds to a 1.8-fold increased risk of T2DM (25, 26). Additionally, sleep disorders such as OSA exacerbate insulin resistance through intermittent hypoxia, with affected patients demonstrating a 2.5-fold higher risk of T2DM than those without OSA (9, 27) (See Figure 1).

## Pathophysiological mechanism of PCOS in T2DM

The pathological interplay between T2DM and PCOS predominantly manifests through three key mechanisms: insulin resistance (IR), hyperandrogenism, and chronic low-grade inflammation. Insulin resistance is a central pathological characteristic common to both conditions, occurring in approximately 70% of PCOS patients and up to 85% of T2DM patients (28). Empirical evidence indicates that the Homeostatic Model Assessment of Insulin Resistance (HOMA-IR) values in PCOS patients are significantly elevated compared to those in healthy controls (21). Hyperandrogenism exacerbates insulin resistance via multiple pathways: androgens can directly impair the insulin signaling cascade by reducing tyrosine phosphorylation of insulin receptor substrate (IRS), and they can also induce adipocyte hypertrophy, leading to increased free fatty acid release, which further intensifies IR (29). Clinical data demonstrate that each unit increase in the free androgen index (FAI) corresponds to a 0.12 increment in HOMA-IR (30).

Chronic low-grade inflammation also plays a pivotal role in the pathological nexus between T2DM and PCOS. Serum C-reactive protein (CRP) levels have been found to be elevated in PCOS patients, though this association is complex and may not be fully independent of adiposity (31). Proinflammatory cytokines such as tumor necrosis factor-alpha (TNF-α) and interleukin-6 (IL-6) contribute to the aggravation of insulin resistance by inhibiting insulin receptor tyrosine kinase activity and simultaneously promoting ovarian androgen synthesis (32). Furthermore, intestinal dysbiosis is implicated in this pathological interaction; specifically, PCOS patients exhibit a reduction in Bacteroidetes and an increase in Firmicutes within their gut microbiota. This dysbiotic state exacerbates inflammatory responses and insulin resistance through mechanisms involving endotoxemia (33).

Traditional Chinese medicine exerts multitargeted and multimodal regulatory effects on the pathological mechanisms underlying the coexistence of T2DM and PCOS. Liuwei Dihuang Pill, a classical kidney-tonifying formulation, has been shown to ameliorate insulin resistance in animal models by modulating the PI3K/Akt signaling pathway, as evidenced by a reduction in IRS-1 (S307) phosphorylation and an increase in PI3Kp85α phosphorylation (34). Concurrently, this formula upregulates Cyp19a1 mRNA expression in ovarian tissue, thereby enhancing estrogen synthesis and reducing testosterone levels (34). Berberine, a widely studied TCM monomer, improves glucose and lipid metabolism by activating the AMP-activated protein kinase (AMPK) pathway. Its hypoglycemic efficacy is comparable to that of metformin, yet it is associated with a 50% lower incidence of adverse gastrointestinal effects (35).

Moreover, TCM effectively modulated inflammatory responses and gut microbiota composition. Clinical studies have reported that Chinese herbal compounds reduce serum CRP levels and TNF-α levels in PCOS patients (36). Additionally, TCM interventions can restore gut microbial balance by increasing the relative abundance of Bacteroidetes and decreasing Firmicutes, thereby elevating the Bacteroidetes/Firmicutes ratio from 0.32 to 0.58 (33). This modulation of the microbiota may be mediated through enhanced production of short-chain fatty acids (SCFAs), with fecal concentrations of acetic acid, propionic acid, and butyric acid increasing by 25%, 30%, and 35%, respectively (37).

Current research has focused on epigenetics, the gut microbiota, and metabolomics in the context of T2DM combined with PCOS. Epigenetic analyses have revealed significant alterations in DNA methylation patterns within the ovarian tissues of PCOS patients, notably a 2- to 3-fold increase in the methylation of genes implicated in insulin signaling pathways, such as IRS1 and PIK3R1 (38). Investigations into intestinal microbiota have identified disease-associated shifts, including decreased abundance of *Bifidobacterium* species and increased *Desulfovibrio* species in PCOS patients, with these microbial changes correlating significantly with insulin resistance and hyperandrogenism (33). Metabolomic studies have demonstrated elevated serum levels of branched-chain amino acids (BCAAs) in individuals with PCOS and T2DM, with leucine, isoleucine, and valine concentrations rising by 22%, 25%, and 20%, respectively (39).

**FIGURE 2:**
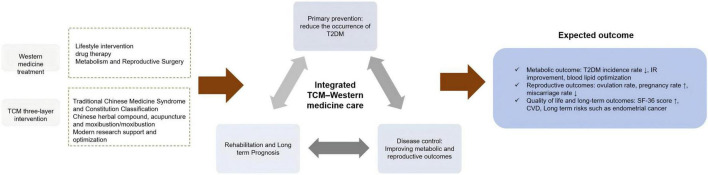
Metabolic axes and endocrine axes and their multi-omics interactions. This figure illustrates the metabolic and endocrine axes linking insulin resistance, high androgen levels, and inflammation in polycystic ovary syndrome (PCOS) and type 2 diabetes mellitus (T2DM). The left section describes the interaction between insulin resistance (IR) and high androgen production, which leads to fat cell hypertrophy and increased free fatty acids, further aggravating IR. The role of inflammation, both high and low degree, in exacerbating IR and androgen production is also depicted. The right section showcases the multiple omics levels involved in PCOS and T2DM, including genetic (shared gene loci like FTO, LHCGR, TCF7L2), epigenetic (methylation of genes such as PDX1, IRS1), gut microbiota, and metabolomics (BCAA levels), which all contribute to the development and exacerbation of the disease. Potential therapeutic targets such as PI3K/Akt signaling, AMPK pathway, and inflammatory factors are also suggested.

Future research directions encompass three primary areas: first, the development of targeted therapeutics addressing shared pathological mechanisms, such as TCM-derived monomers targeting the PI3K/Akt pathway; second, exploration of intestinal microbiota transplantation as a therapeutic modality; and third, integration of multi-omics approaches to elucidate the molecular regulatory networks underlying these diseases. These investigative avenues hold promise for advancing precision medicine strategies for the management of T2DM concomitant with PCOS (See Figure 2).

## Genetic study of PCOS in T2DM

Polycystic ovary syndrome and T2DM are both polygenic hereditary disorders, exhibiting heritability estimates of approximately 70% and 60%, respectively, and share several susceptibility loci (24, 40). Genome-wide association studies (GWAS) have identified that genes implicated in PCOS susceptibility, including FTO, LHCGR, and THADA, are also associated with an increased risk of T2DM. Specifically, the A allele of the FTO rs9939609 variant not only elevates obesity risk but also modulates the hypothalamic appetite center and insulin signaling pathways. Furthermore, the G allele of LHCGR rs2293275 is significantly correlated with hyperandrogenism and insulin resistance in individuals with PCOS, conferring a 2.1-fold increased risk of developing T2DM (41).

Epigenetic investigations have demonstrated that intrauterine exposure to hyperandrogenism can influence insulin sensitivity in the offspring via DNA methylation modifications. For instance, female rat offspring exposed to hyperandrogenism during gestation exhibit a PCOS-like phenotype in adulthood, accompanied by increased methylation of the PDX1 promoter in pancreatic islet beta cells, which leads to impaired insulin secretion (38). Additionally, aberrant expression of non-coding RNAs, such as miR-122 and miR-223, has been observed in T2DM patients with PCOS. MiR-122 facilitates hepatic lipogenesis through targeted regulation of SREBP-1c, whereas miR-223 exacerbates insulin resistance by suppressing IRS1 expression (42). Notably, genetic factors exhibit racial variability; for example, the risk of T2DM among PCOS patients of South Asian descent is 1.8 times greater than that observed in Caucasian patients, potentially attributable to differences in the allele frequencies of susceptibility genes such as TCF7L2 and KCNQ1 (5, 13).

## Diagnosis

The diagnosis of T2DM concomitant with PCOS necessitates the fulfillment of the independent diagnostic criteria for each condition, along with the exclusion of alternative differential diagnoses. T2DM diagnosis adheres to the World Health Organization (WHO) 2019 guidelines and is defined by fasting plasma glucose levels ≥ 7.0 mmol/L, 2-h postprandial glucose ≥ 11.1 mmol/L, or glycated hemoglobin (HbA1c) ≥ 6.5% (1). According to the 2023 International Evidence-based Guideline, PCOS diagnosis in adults is based on the revised consensus Rotterdam criteria, updated to evidence-based criteria, requiring two of the following after exclusion of related disorders: (1) ovulatory dysfunction; (2) clinical or biochemical hyperandrogenism; and (3) polycystic ovarian morphology on ultrasound or elevated AMH levels (7). Additionally, conditions such as congenital adrenal hyperplasia, thyroid dysfunction, and hyperprolactinemia must be excluded (2, 3).

In clinical practice, a comprehensive evaluation of metabolic and reproductive parameters is essential. For suspected cases, assessments should include fasting blood glucose, HbA1c, and OGTT to evaluate glucose metabolism, along with the measurement of six sex hormones: total testosterone, free testosterone, luteinizing hormone (LH), follicle-stimulating hormone (FSH), estradiol (E2), sex hormone-binding globulin (SHBG), and pelvic ultrasonography (6). In adolescents, PCOS diagnosis should be made cautiously. According to the 2023 guideline, both hyperandrogenism and ovulatory dysfunction are required for diagnosis, whereas ultrasound and AMH are not recommended because of poor specificity in this age group (7). Adolescents with suggestive features who do not yet meet diagnostic criteria may be considered at increased risk and reassessed over time. Furthermore, patients presenting with both T2DM and PCOS frequently exhibit NAFLD, warranting the evaluation of hepatic steatosis severity via liver function tests, ultrasonography, or FibroScan (8).

Pelvic ultrasonography is an important imaging modality used for the diagnosis of PCOS. Transvaginal ultrasound (TVUS) demonstrates superior diagnostic accuracy compared to transabdominal ultrasound, effectively delineating ovarian volume (≥10 mL) and follicle number (≥12 per ovary, diameter 2–9 mm), with a sensitivity exceeding 90% (2, 43). Additionally, ultrasonographic assessment of endometrial thickness is critical, given that patients with T2DM and PCOS exhibit a 3- to 4-fold increased risk of endometrial carcinoma, necessitating regular monitoring; endometrial thickness ≥ 5 mm requires further investigation (44).

Metabolic imaging evaluations include liver ultrasonography, abdominal computed tomography (CT), magnetic resonance imaging (MRI), and dual-energy X-ray absorptiometry (DXA). Liver ultrasonography detected NAFLD with approximately 80% sensitivity and 90% specificity (8). Abdominal MRI quantitatively measures visceral adipose tissue, which is elevated by 30–50% in patients with both T2DM and PCOS compared to those with PCOS alone (45). DXA assesses bone mineral density (BMD), which is typically 5%–10% higher in PCOS patients than in healthy controls; however, concomitant T2DM may negate this advantage, resulting in reduced BMD (46). Emerging modalities such as magnetic resonance spectroscopy (MRS) offer a quantitative evaluation of hepatic fat content with an accuracy exceeding 95%, facilitating early NAFLD detection (43).

Glucose metabolism assessment includes fasting plasma glucose, HbA1c, and OGTT, with the latter regarded as the gold standard for diagnosing impaired glucose tolerance (IGT). The prevalence of IGT among PCOS patients ranges from 20% to 30%, which is significantly higher than that observed in the general population (6, 19). Insulin resistance can be quantified using indices such as HOMA-IR and the quantitative insulin sensitivity check index (QUICKI); a HOMA-IR value ≥ 2.5 is indicative of insulin resistance. The mean HOMA-IR in PCOS patients is 3.2 ± 1.5, approximately two to three times higher than that in healthy women (47).

**FIGURE 3:**
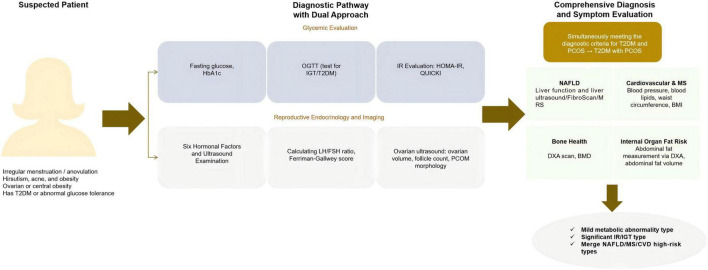
Dual evaluation pathway for polycystic ovary syndrome (PCOS) and type 2 diabetes mellitus (T2DM) diagnosis. This figure presents a diagnostic pathway for diagnosing PCOS in women with T2DM. The first part involves the identification of suspected patients with irregular menstruation, anovulation, hirsutism, acne, obesity, and ovarian or central obesity. In these patients, glycemic evaluations are conducted, including fasting glucose, HbA1c, and OGTT to screen for T2DM or impaired glucose tolerance (IGT). The second part includes reproductive endocrinology and imaging, with tests like the six hormonal factors, ultrasound examination, and the calculation of the LH/FSH ratio and Ferriman-Gallwey score. Ovarian ultrasound is used to assess ovarian volume, follicle count, and PCOM morphology. The comprehensive evaluation also involves assessing comorbidities such as NAFLD, cardiovascular risks, and bone health.

The evaluation of sex hormones should encompass total and free testosterone, LH, FSH, and SHBG. Free testosterone serves as the most reliable marker of hyperandrogenism, with mean levels in PCOS patients measuring 4.8 ± 1.2 pg/mL, two to three times greater than those in healthy females (1, 4). An LH/FSH ratio ≥ 2.5 suggests ovarian-derived hyperandrogenism, exhibiting a sensitivity of approximately 70% (2). Metabolic assessments should also include lipid profiles, including total cholesterol (TC), low-density lipoprotein cholesterol (LDL-C), high-density lipoprotein cholesterol (HDL-C), and triglycerides (TG), liver function tests [alanine aminotransferase (ALT), aspartate aminotransferase (AST)], and inflammatory markers such as high-sensitivity C-reactive protein (hsCRP). PCOS patients demonstrate TG levels elevated by 30%–50% and HDL-C levels reduced by 20%–30% relative to healthy controls (13). Moreover, hsCRP concentrations increase two- to three-fold and correlate positively with the degree of insulin resistance (29) (See Figure 3).

## Therapeutic strategies for T2DM with PCOS

### Lifestyle intervention in patients with T2DM and PCOS

Lifestyle modification remains the primary therapeutic approach for managing T2DM in patients with PCOS, with a focus on weight regulation and metabolic improvement. Dietary interventions emphasizing low-glycemic index (GI) foods have been shown to reduce postprandial glycemic fluctuations and improve insulin sensitivity. For example, a randomized controlled trial demonstrated that a 12-week low-GI dietary regimen was associated with a meaningful reduction in insulin resistance, as reflected by decreased HOMA-IR, along with a concurrent reduction in circulating testosterone levels among patients with PCOS (26). In addition, adherence to a Mediterranean dietary pattern has been associated with a lower cardiovascular risk profile and improvements in lipid metabolism. A 12-week Mediterranean diet intervention was reported to reduce triglyceride levels and increase HDL-C concentrations (26). Caloric restriction, typically involving a daily energy deficit of 500–750 kcal, is commonly associated with modest but clinically significant weight loss, which contributes to improvements in ovulatory function and glucose metabolism. Notably, even moderate weight reduction has been linked to restoration of menstrual regularity in a substantial proportion of patients and a reduced risk of progression to T2DM (25, 48).

Regarding physical activity, a combination of aerobic exercise (e.g., brisk walking and swimming) and resistance training yielded optimal outcomes. Engaging in 150 min/week of moderate-intensity aerobic exercise can lower HbA1c levels by 0.5%–1.0% and improve insulin sensitivity (25). Resistance training performed two to three times weekly contributes to increased muscle mass, elevated basal metabolic rate, and further metabolic improvements (49). Behavioral strategies, such as cognitive behavioral therapy (CBT), have been shown to enhance adherence to lifestyle modifications, and one study indicated that integrating CBT with dietary and exercise interventions improved weight loss maintenance by 20%–30% (49). Furthermore, interventions targeting sleep disorders, including OSA management, can reduce nocturnal cortisol secretion and improve insulin resistance. Continuous positive airway pressure (CPAP) therapy for OSA has been demonstrated to decrease HOMA-IR (9).

### Drug therapy for T2DM with PCOS

When lifestyle modifications prove insufficient or glycemic control remains suboptimal, pharmacological intervention should be initiated. Metformin, administered at a dosage of 1,500–2,000 mg/day, is the preferred therapeutic agent for T2DM. It has been demonstrated to lower fasting blood glucose levels by approximately 1.0–1.5 mmol/L and ameliorate insulin resistance and hyperandrogenism. A meta-analysis indicated that metformin reduces total testosterone concentrations and free testosterone levels in patients with PCOS (50, 51). For individuals who exhibit intolerance to metformin or an inadequate therapeutic response, glucagon-like peptide-1 (GLP-1) receptor agonists such as liraglutide and semaglutide may be employed adjunctively. Liraglutide has been shown to decrease glycated hemoglobin (HbA1c), reduce body weight and enhance ovarian function (52, 53).

Management of PCOS-associated manifestations encompasses several targeted approaches: (1) hyperandrogenism is primarily addressed with combined oral contraceptives (COCs), including formulations containing ethinyl estradiol and cyproterone acetate, which can reduce total testosterone levels by nearly 30% and alleviate symptoms such as hirsutism and acne (50); (2) ovulatory dysfunction is treated preferentially with letrozole (2.5–5 mg daily for 3–7 days during the menstrual cycle) in patients desiring fertility, yielding ovulation rates of 70%–80% and live birth rates that are 15%–20% higher compared to clomiphene citrate (50, 54); (3) metabolic syndrome components may be managed with statins, such as atorvastatin, which can lower LDL-C and improve insulin sensitivity (55). It is imperative to monitor potential drug interactions; for instance, when metformin is co-administered with COCs, blood glucose levels should be closely monitored to prevent hypoglycemic episodes (56).

### Surgical treatment of T2DM with PCOS

Surgical intervention is primarily indicated for patients with severe obesity [body mass index (BMI) ≥ 35 kg/m^2^] or those who exhibit an inadequate response to pharmacological treatment (57). Metabolic surgeries, including laparoscopic sleeve gastrectomy (LSG) and Roux-en-Y gastric bypass (RYGB), have been demonstrated to result in substantial weight loss within 1 year postoperatively, along with improvements in glucose metabolism (58). Studies have reported remission rates of approximately 60%–80% within 2 years after bariatric surgery, although estimates vary depending on study population and definitions of remission. Furthermore, remission rates for PCOS symptoms such as menstrual irregularities and hyperandrogenism range from 50% to 60% (59). Additionally, metabolic surgery is associated with a significant reduction in the risk of endometrial cancer, with incidence rates 5 years post-surgery being 80%–90% lower than that in non-surgical cohorts.

Procedures specifically targeting PCOS, such as laparoscopic ovarian drilling (LOD), are considered in women with anovulatory infertility who are resistant to pharmacological ovulation induction. LOD reduces ovarian androgen production through stromal ablation, thereby improving ovulatory function. Evidence from a Cochrane systematic review indicates that live birth rates following LOD range from approximately 28% to 40%, although comparative analyses suggest that LOD may not improve live birth rates compared with medical ovulation induction alone, and the overall effect remains uncertain (60). It is important to consider potential surgical complications, including malnutrition following metabolic surgery (occurring in 5%–10% of cases) and premature ovarian failure after LOD (1%–2%). Regarding surgical timing, metabolic surgery is advised during the early reproductive years to mitigate adverse effects on fertility, whereas LOD is recommended only after unsuccessful pharmacological ovulation induction to prevent premature surgical intervention.

## Basic theory of TCM on T2DM with polycystic ovary syndrome

Type 2 diabetes mellitus and PCOS are classified as TCM under the categories of “consumptive thirst,” “amenorrhea,” and “infertility.” Their fundamental pathogenesis is conceptualized as kidney deficiency, which is the root cause, accompanied by phlegm dampness and blood stasis as symptomatic manifestations. Contemporary research indicates that the prevalence of T2DM among individuals with PCOS is markedly elevated, approximately fourfold higher than that observed in the general population (57). From a TCM perspective, kidney deficiency constitutes the shared pathological foundation of both conditions, given the kidney’s role in storing essence, regulating reproduction, and managing water metabolism. Kidney deficiency disrupts the Chong and Ren meridians, leading to menstrual irregularities and ovulatory dysfunction. Impaired kidney function results in insufficient essence consolidation, thereby contributing to glucose metabolic disturbances (34). Clinical evidence reveals that more than half of PCOS patients exhibit varying degrees of IR, which, within TCM syndrome differentiation, is predominantly associated with phlegm dampness arising from kidney deficiency (61).

Further investigations have elucidated that the interplay between the gut microbiota and iron metabolism may participate in the pathophysiological mechanisms underlying both disorders. Dysbiosis of intestinal flora can induce chronic low-grade inflammation, thereby facilitating the onset and progression of IR. Concurrently, aberrant iron metabolism exacerbates glucose metabolic dysfunction by impairing insulin signaling pathways (33). Epidemiological data demonstrate that exposure to organochlorine pesticides (OCPs) increases the risk of PCOS by 2.3 times, with affected individuals exhibiting a heightened propensity to develop T2DM (62). Within the TCM framework, OCPs are conceptualized as external toxins that compromise spleen and kidney functions, thereby intensifying phlegm dampness and blood stasis. Moreover, elevated serum levels of follistatin (FST) have been significantly correlated with increased risks of PCOS and T2DM, with odds ratios of 1.129 and 1.103, respectively (63). This observation aligns with the TCM theory of “Qi and blood imbalance,” as FST, which functions as a cytokine, reflects disruptions in the circulation of Qi and blood.

The therapeutic approach of TCM for managing T2DM concomitant with PCOS is grounded in the principles of holistic treatment and syndrome differentiation. Clinical studies have demonstrated that TCM formulations exert multifaceted effects: they modulate gut microbiota composition by reducing the Firmicutes/Bacteroides ratio and enhancing intestinal barrier integrity (36); concurrently, they attenuate inflammatory responses, as evidenced by a reduction in serum CRP levels (64). A nationwide cohort study further substantiated that TCM intervention significantly decreased the incidence of T2DM among PCOS patients, with a hazard ratio of 0.31 (64), thereby corroborating the TCM concept of “preventive treatment of disease” through early therapeutic intervention to impede disease progression.

Modern pharmacological investigations have provided empirical support for the scientific validity of TCM theory. Liuwei Dihuang Pill, a classical kidney-tonifying formula, has been shown to enhance insulin sensitivity via activation of the PI3K/Akt signaling pathway. In animal models, treatment resulted in reductions in IRS-1 (S307) phosphorylation and increase in PI3Kp85α phosphorylation (34). Additionally, this formula upregulated Cyp19a1 mRNA expression in ovarian tissue, promoted estrogen synthesis, and decreased testosterone levels (34), thereby offering molecular biological evidence for the TCM principle of “kidney governing reproduction.” Furthermore, TCM interventions have been observed to regulate adipokine secretion, increase adiponectin levels and reduce leptin levels (36), consistent with the TCM description of fat metabolism disorders attributed to phlegm dampness accumulation.

## Treatment of T2DM with PCOS by TCM

### Clinical practice of T2DM with PCOS treated by TCM

The clinical application of TCM in managing T2DM concomitant with PCOS primarily encompasses three modalities: herbal compound formulation, acupuncture, and lifestyle interventions. The principle of syndrome differentiation and individualized treatment is central to the use of TCM herbal compounds. For instance, Liuwei Dihuang Pill is frequently prescribed for patients with kidney deficiency, Cangfu Daotan Pill is utilized for those with phlegm-dampness syndrome, Taohong Siwu Decoction is indicated for blood stasis, and Xiaoyao Powder is commonly administered to patients presenting with liver depression (64). A cohort study involving 342 patients demonstrated that TCM interventions were associated with a 69% reduction in the incidence of T2DM, reflected by a hazard ratio (HR) of 0.31 (64). Further subgroup analysis revealed that compound preparations containing Paeonia lactiflora exerted a more pronounced protective effect with an OR of 0.28 (64).

Acupuncture and moxibustion primarily exert their therapeutic effects by modulating endocrine and metabolic functions. A randomized controlled trial reported that acupuncture reduced HOMA-IR by 14.7% in patients with PCOS compared to a 25.0% reduction observed in the metformin treatment group. Although acupuncture demonstrated slightly less efficacy than metformin in lowering blood glucose levels, it was associated with a significantly lower incidence of adverse gastrointestinal effects (65). Additionally, acupuncture contributes to the improvement of menstrual regularity, with the rate of regular menstrual cycles increasing from 32% to 68% post-treatment (65).

Lifestyle interventions, grounded in the TCM concept of “preventive treatment of disease,” incorporate dietary regulation, physical exercise, and emotional management. Empirical evidence indicates that a low glycemic index (GI) diet combined with TCM interventions can lead to a 5%–8% reduction in patient body weight, concomitant with enhanced insulin sensitivity (64).

### Individualized design of TCM treatment

The formulation of personalized treatment protocols for TCM in managing T2DM concomitant with PCOS primarily relies on syndrome differentiation and constitutional identification. Regarding syndrome differentiation and classification, therapeutic strategies for patients exhibiting kidney deficiency emphasize kidney tonification and essence replenishment, commonly employing herbs such as prepared rehmannia root, yams, and Cornus officinalis. For patients characterized by phlegm dampness, treatment focuses on resolving phlegm and eliminating dampness, utilizing agents such as *Atractylodes*, *Poria cocos*, and tangerine peel. In cases of blood stasis, the therapeutic approach centers on promoting blood circulation and removing stasis, with frequently used herbs, including peach kernel, safflower, and angelica. Patients presenting with liver depression are treated by soothing the liver and regulating qi, often with *Bupleurum*, *Paeonia lactiflora*, and Fructus Aurantii (64).

Concerning constitution identification, individuals with a mild constitution may receive conventional therapy, whereas those with qi deficiency are administered qi-invigorating herbs, such as *Astragalus membranaceus* and *Codonopsis pilosula*. Patients exhibiting yin deficiency are treated with yin-nourishing herbs such as *Ophiopogon japonicus* and *Polygonatum odoratum*, while those with yang deficiency receive yang-warming herbs, including Aconite and Cinnamomum cassia (66).

The integration of modern technological approaches offers novel avenues for designing individualized treatment regimens. Metabonomic analyses have revealed significant variations in metabolic profiles among patients with distinct syndrome types; specifically, serum amino acid levels are markedly reduced in individuals with kidney deficiency, whereas serum lipid levels are significantly elevated in those with phlegm dampness (66). Leveraging these metabolic distinctions, researchers have developed a metabonomics-based syndrome differentiation model that demonstrates an accuracy exceeding 80% (66). Furthermore, investigations of the intestinal microbiota provide an additional foundation for personalized treatment design. For instance, *Bifidobacterium* populations were diminished in the intestines of patients with kidney deficiency, whereas *Desulfovibrio* levels were increased in those with phlegm dampness. Consequently, tailored Chinese medicinal interventions can be implemented based on these microbial characteristics (33).

### Evaluation and optimization of the curative effect of TCM

Evaluation of the therapeutic efficacy of TCM in the management of T2DM concomitant with PCOS primarily encompasses three domains: amelioration of clinical signs and symptoms, enhancement of metabolic parameters, and improvement in quality of life. Regarding clinical symptomatology, TCM interventions have been shown to increase menstrual regularity rates from 32% to 68% and elevate pregnancy rates among infertile patients from 15% to 35% (64). In terms of metabolic indices, TCM treatment reduced the HOMA-IR by 14.7%, testosterone levels by 29%, and fasting blood glucose levels by 12% (34, 65). Regarding quality of life, patients’ scores on the SF-36 questionnaire improved significantly, increasing from 52 points pre-treatment to 75 points post-treatment (64).

**FIGURE 4:**
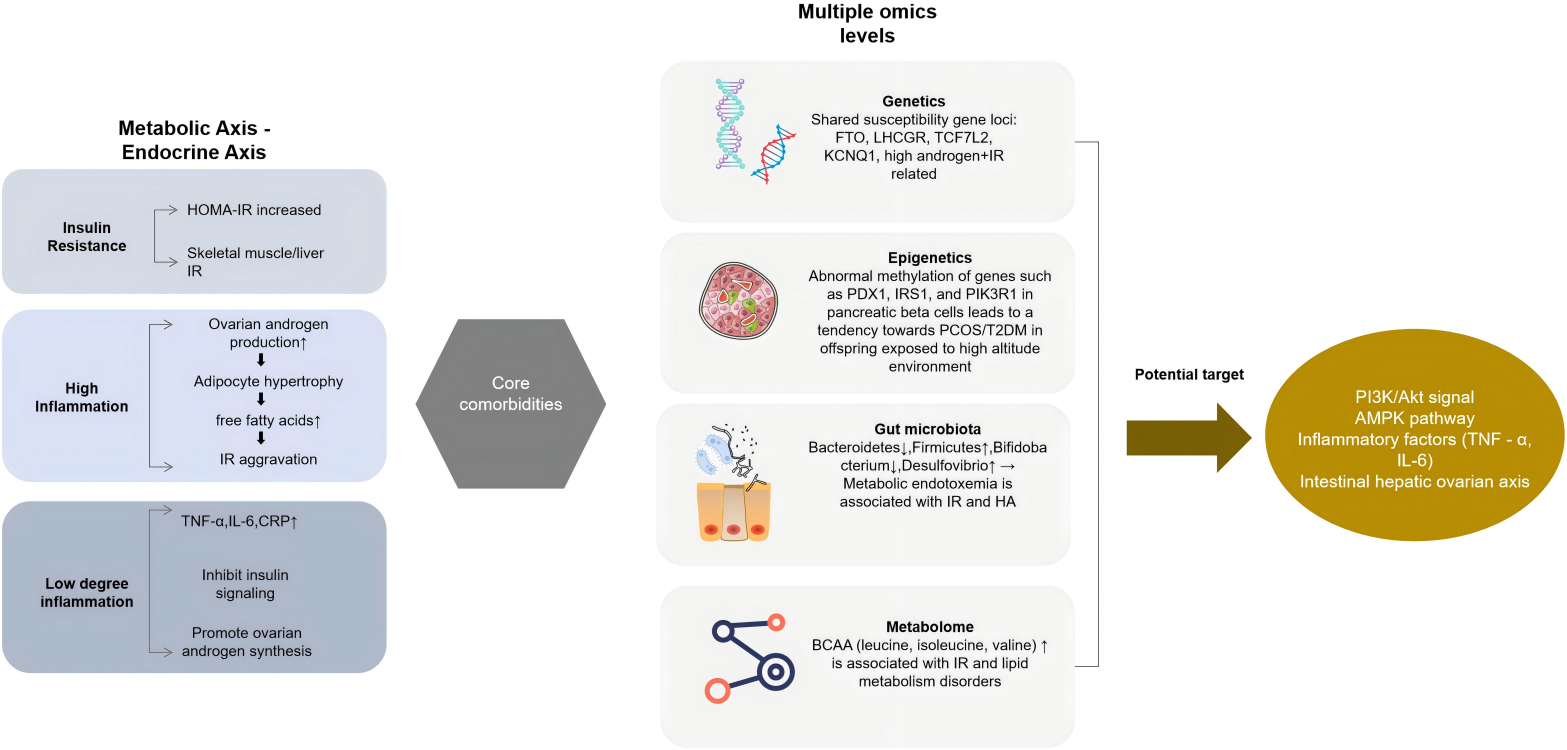
Integrated traditional Chinese and Western medicine comprehensive management framework. This figure illustrates the integrated care approach for women with polycystic ovary syndrome (PCOS) and type 2 diabetes mellitus (T2DM). The first part shows Western medicine treatments, including lifestyle interventions, drug therapy, and metabolic and reproductive surgery. The second part illustrates traditional Chinese medicine (TCM) interventions, including syndrome differentiation, herbal compounds, acupuncture, moxibustion, and modern research support. The combined TCM–Western medicine approach aims to reduce the occurrence of T2DM (primary prevention), improve metabolic and reproductive outcomes (disease control), and enhance rehabilitation and long-term prognosis. The expected outcomes include a reduction in T2DM incidence, improved insulin resistance, better reproductive outcomes, and improved quality of life.

Efforts to optimize therapeutic efficacy focus on three principal strategies: first, refinement of TCM compound formulations, such as identifying core pharmacological agents through network pharmacology approaches; second, enhancement of drug delivery methods, exemplified by the application of nanotechnology to increase bioavailability; and third, integration of precise treatment regimens combining TCM with contemporary medical practices. Network pharmacology analyses have identified *Rehmannia glutinosa*, *Dioscorea opposita*, and *Cornus officinalis* as key constituents of the Liuwei Dihuang Pill, with shared molecular targets, including IRS1 and PIK3R1 (34). Nano-biological advancements have demonstrated that berberine nanoparticles can achieve a 3- to 5-fold increase in bioavailability (67). Furthermore, emerging therapeutic modalities involving modulation of the gut microbiota are under investigation. Current evidence suggests that intestinal flora and iron metabolism interact in the development and progression of metabolic syndrome-related comorbidities, including T2DM and PCOS, which may provide a potential basis for microbiota-related therapeutic strategies (33) (See Figure 4).

## Controversial points and future prospects of T2DM with PCOS

### Controversies and challenges of TCM

The debate surrounding the use of TCM in the treatment of T2DM complicated by PCOS primarily centers on three key issues: first, the paucity of robust evidence grounded in evidence-based medicine, the ambiguous mechanisms underlying its therapeutic effects, and the absence of standardized quality control measures. Although some randomized controlled trials have been conducted, these studies typically involve small sample sizes and lack multi-center, large-scale investigations (8). Regarding the mechanisms of action, while certain studies have identified specific pathways through which TCM exerts its effects, the comprehensive regulatory framework involving its multicomponent and multitarget nature remains inadequately elucidated (34). Concerning quality control, variables such as the origin and processing methods of TCM significantly influence therapeutic efficacy; however, no unified standards for quality assurance have been established (68).

The challenges confronting the integration and advancement of TCM in this context can be categorized into four main areas: (1) standardization and objectification of TCM syndrome differentiation, (2) enhancement of TCM bioavailability, (3) effective integration with contemporary medical treatment protocols, and (4) cultivation of interdisciplinary professionals proficient in both TCM and modern medicine. Although objective diagnostic tools, such as tongue and pulse diagnostic instruments, have been developed to aid syndrome differentiation, these technologies require further refinement to improve reliability and validity (69). In terms of bioavailability, modern pharmaceutical technologies, including nanotechnology, have the potential to enhance drug absorption, albeit at a considerable financial cost (67). Regarding integrated treatment approaches, further research is necessary to determine the optimal therapeutic regimens tailored to individual patient profiles (64). Finally, addressing the need for interdisciplinary expertise necessitates the establishment of comprehensive educational frameworks that bridge the traditional and modern medical disciplines (68).

### Future research directions of T2DM with PCOS

Future research on the comorbidity of T2DM and PCOS should focus on these five primary areas. First, conducting multicenter, large-sample, randomized controlled trials is essential to generate high-quality evidence supporting the efficacy of TCM treatments. Second, employing multi-omics technologies to elucidate the molecular mechanisms underlying the disease and to identify the therapeutic targets of TCM is crucial. Third, the development of novel therapeutic agents based on the TCM theory should be pursued. Fourth, establishing an integrated diagnostic and treatment framework that combines TCM and Western medicine is necessary. Fifth, health economics research should be conducted to evaluate the cost-effectiveness of TCM interventions. Within multicenter trials, Standardization of diagnostic criteria, treatment protocols, and outcome measures is imperative (64). Regarding multi-omics investigations, the integration of genomics, transcriptomics, proteomics, and metabolomics data is required to map the molecular networks involved in a disease (38). In drug development, screening for core compounds based on TCM principles and creating novel drugs with independent intellectual property rights are recommended (34). For the integration of medical systems, the formulation of clinical guidelines that harmonize TCM and Western medicine practices is required (68). Lastly, health economic analyses should focus on assessing the long-term cost-effectiveness of TCM therapies (64).

### Prospect of TCM in comprehensive treatment

The potential role of TCM in the comprehensive management of T2DM coexisting with PCOS can be delineated across the three primary dimensions. First, TCM serves as an adjunctive therapy aimed at enhancing metabolic parameters and alleviating clinical symptoms. Second, it functions as a preventive strategy to decrease the incidence of the disease. Third, TCM contributes to rehabilitation efforts focused on improving the patients’ quality of life. Regarding adjunctive therapy, TCM can be integrated with conventional pharmacological agents, such as metformin and contraceptives, to augment therapeutic efficacy and mitigate adverse effects (64). In the preventive context, TCM may reduce the onset of T2DM in individuals with PCOS by modulating the physical constitution and promoting healthier lifestyle practices (64). Non-pharmacological TCM interventions, including acupuncture and massage, have been shown to enhance the patients’ quality of life (69).

The application of TCM in treating T2DM complicated by PCOS is anticipated to evolve along three key trajectories. The first involves a shift from empirical practice to evidence-based medicine, supported by an increasing number of rigorous clinical studies (64). The second trajectory emphasizes a transition from macroscopic to microscopic syndrome differentiation, facilitated by the integration of modern molecular biology techniques to achieve objectivity and standardization in syndrome classification (66). The third trend entails a shift from monotherapy to integrated treatment approaches, establishing a diagnostic and therapeutic framework that combines traditional Chinese and Western medical practices to deliver personalized treatment regimens (68). Collectively, these developments are expected to advance the role of TCM in the management of T2DM with PCOS, ushering in a new era of clinical applications.
