# Supplementary material for: Exploring the comorbidity of type 2 diabetes mellitus and polycystic ovary syndrome: treatment with traditional Chinese medicine
Source: Front Med (Lausanne). 2026 May 25;13:1764278. doi: 10.3389/fmed.2026.1764278 (PMC13243291; doi:10.3389/fmed.2026.1764278)
Supplement: Supplementary file 1 [file Supplementary_file_1.docx]

**Supplementary Material: Methods**

**Literature Search Strategy**

A literature search was conducted to identify relevant studies addressing the epidemiology, pathophysiology, diagnosis, and management of type 2 diabetes mellitus (T2DM) with polycystic ovary syndrome (PCOS), as well as the role of traditional Chinese medicine (TCM) in this comorbidity. Electronic databases including PubMed, Web of Science, Embase, and CNKI (China National Knowledge Infrastructure) were searched.

The search strategy combined Medical Subject Headings (MeSH) terms and free-text keywords, including but not limited to: “polycystic ovary syndrome”, “PCOS”, “type 2 diabetes mellitus”, “T2DM”, “insulin resistance”, “hyperandrogenism”, “metabolic syndrome”, “gut microbiota”, “epigenetics”, “traditional Chinese medicine”, and “integrative medicine”. The reference lists of the relevant articles and reviews were manually screened to identify additional eligible studies.

**Eligibility Criteria**

Studies were considered eligible if they met the following criteria: investigated T2DM, PCOS, or their comorbidity; reported on epidemiology, pathophysiology, genetic or epigenetic mechanisms, diagnosis, or treatment strategies; included original research (e.g., randomized controlled trials, cohort studies, case–control studies), systematic reviews, or meta-analyses; and published in English. Studies were excluded if they were conference abstracts without full text; lacked sufficient discussions on our review topic; and focused on unrelated endocrine or metabolic conditions.

**Study Selection**

Relevant studies were screened based on titles and abstracts, followed by full-text evaluation. Key information was extracted, including study design, population characteristics, primary outcomes, and major findings related to T2DM–PCOS comorbidity. Priority was given to high-quality evidence, including systematic reviews, meta-analyses, and large-scale cohort studies. Mechanistic studies, including molecular and animal research, were included where relevant to support pathophysiological interpretations.

**Integration of Traditional Chinese Medicine Evidence**

Evidence related to TCM was derived from clinical studies, pharmacological investigations, and mechanistic research. Due to variabilities in study qualities and designs, TCM-related findings were interpreted cautiously and presented as complementary to conventional medical evidence.
